# Dynamic functions of GABA signaling during granule cell maturation

**DOI:** 10.3389/fncir.2012.00113

**Published:** 2013-01-08

**Authors:** Cristina V. Dieni, Jessica H. Chancey, Linda S. Overstreet-Wadiche

**Affiliations:** Department of Neurobiology, University of Alabama at BirminghamBirmingham, AL, USA

**Keywords:** dentate gyrus, adult neurogenesis, interneuron, parvalbumin, neurogliaform, neural stem cell, neuroprogenitor, activity-dependent

## Abstract

The dentate gyrus is one of the few areas of the brain where new neurons are generated throughout life. Neural activity influences multiple stages of neurogenesis, thereby allowing experience to regulate the production of new neurons. It is now well established that GABA_A_ receptor-mediated signaling plays a pivotal role in mediating activity-dependent regulation of adult neurogenesis. GABA first acts as a trophic signal that depolarizes progenitors and early post mitotic granule cells, enabling network activity to control molecular cascades essential for proliferation, survival and growth. Following the development of glutamatergic synaptic inputs, GABA signaling switches from excitatory to inhibitory. Thereafter robust synaptic inhibition enforces low spiking probability of granule cells in response to cortical excitatory inputs and maintains the sparse activity patterns characteristic of this brain region. Here we review these dynamic functions of GABA across granule cell maturation, focusing on the potential role of specific interneuron circuits at progressive developmental stages. We further highlight questions that remain unanswered about GABA signaling in granule cell development and excitability.

## Introduction

The dentate gyrus (DG) contains a pool of neuronal stem cells that generates new dentate granule cells (GCs) throughout the life of mammals, including humans (Eriksson et al., 1998). These adult-generated neurons become synaptically integrated into the existing circuitry and participate in normal hippocampal function. While the role of adult neurogenesis in behavior is not fully understood, treatments that enhance neurogenesis, such as environmental enrichment, exercise and electrical stimulation, are well known to enhance cognitive performance whereas treatments that reduce neurogenesis typically impair performance [reviewed by Deng et al. (2010)]. The DG has long been associated with the computational task of pattern separation, i.e., the ability to transform a set of similar inputs into a more distinct pattern of outputs (Marr, 1971). Interestingly, selective manipulations of neurogenesis reliably affect performance in tasks that involve spatial pattern separation (Clelland et al., 2009; Sahay et al., 2011; Nakashiba et al., 2012), suggesting that ongoing neurogenesis is required for this normal dentate function. Other roles of adult neurogenesis in time encoding and memory resolution have also been proposed (Becker and Wojtowicz, 2007; Deng et al., 2010; Aimone et al., 2011).

**Figure 1:**
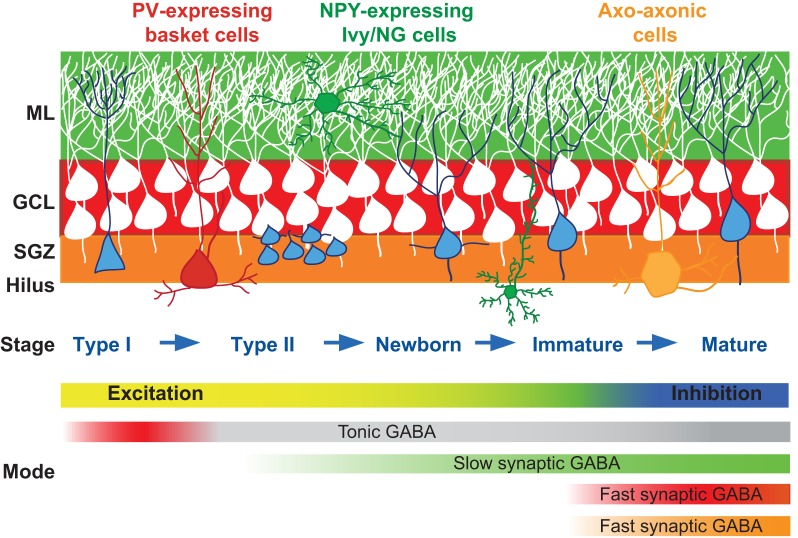
**GABAergic innervation in adult neurogenesis.** Cartoon depiction of the stages of GC maturation highlighting the sequence of interneuron innervation. Progressive stages of GC maturation are indicated by the blue cells. Select interneurons and their axonal targeting regions are indicated by the corresponding shaded areas (red, PV^+^ basket cells; green, Ivy/NG cells; orange, axo-axonal cells). The shift in function of GABA signaling from excitation to inhibition, and the sequence and modes of signaling from interneuron subtypes are indicated in the gradient bars below. The sequence of innervation by numerous other dentate interneuron subtypes [reviewed in Houser (2007)] is not yet known so not included. SGZ, subgranule zone; GCL, granule cell layer; ML, molecular layer.

Adult neurogenesis encompasses the proliferation, differentiation, and maturation of new GCs that are continually added to the dentate. The continuum of neuronal development can be simplified into the stepwise progression of neural stem cells (Type I cells) into progenitors (Type II cells), differentiation of post mitotic newborn neurons, and the synaptic integration of immature GCs, with each stage exhibiting different physiological properties [Figure 1; reviewed by Mongiat and Schinder (2011)]. The absolute number of cells generated each day depends on rodent age and species, with estimates ranging between 2000 and 9000 under basal conditions (Kempermann et al., 1997b; Cameron and McKay, 2001). Adult generated neurons that survive the first few weeks following cell birth are likely to persist long term (Dayer et al., 2003), allowing adult generated neurons to accumulate over time, potentially achieving up to 10% of the total granule cell population (Lagace et al., 2007; Imayoshi et al., 2008). Yet the majority of newly generated cells undergo apoptosis within the week after division (Hayes and Nowakowski, 2002; Sierra et al., 2010), resulting in a population of immature neurons that is a small percentage of the total population of GCs. In young adult mice, it has been estimated that ~10–12 day-old GCs comprise about 3% of the population (Pugh et al., 2011) and 4-week old immature GCs comprise <1% of the population (Kempermann et al., 1997b), whereas in rats there is about twice as many surviving immature GCs (Snyder et al., 2009).

Recent interest has focused on how the population of immature adult generated neurons between 1 and 2 months post-mitosis can make contributions to various hippocampal-dependent behaviors (Kim et al., 2012). During this developmental period immature neurons are fully integrated in hippocampal network, receiving cortical afferents and forming functional output synapses with hilar and CA3 neurons (Esposito et al., 2005; Toni et al., 2007), yet they also retain distinctive immature properties that make them more responsive to synaptic activation. Stimulation of cortical afferents *in vitro* triggers spiking and synaptic plasticity in a greater fraction of immature GCs compared to mature GCs (Schmidt-Hieber et al., 2004; Ge et al., 2007b; Marin-Burgin et al., 2012). This proclivity for excitation is in sharp contrast with the overall sparse population coding evident in the DG *in vivo*, where only a fraction of GCs are activated by sensory stimulation in behaving rodents (Jung and McNaughton, 1993; Chawla et al., 2005; Neunuebel and Knierim, 2012). Thus it has been proposed that the small population of excitable immature GCs can make a significant contribution to overall network function due to preferential recruitment over mature GCs (Kee et al., 2007; Snyder et al., 2009; Alme et al., 2010; Marin-Burgin et al., 2012), but see also (Stone et al., 2011).

The realization that adult neurogenesis is highly regulated by stimuli like exercise and environmental enrichment contributed to its widespread acceptance as a physiologically relevant phenomenon (Kempermann et al., 1997a; van Praag et al., 1999). Each process underlying neurogenesis from stem cell quiescence through synaptic integration is regulated by a surprisingly large number of physiological and pathological stimuli (Ming and Song, 2011). The profound extrinsic regulation of adult neurogenesis identified in animal models represents a dramatic form of plasticity in the adult brain and a potential therapeutic target for human conditions. Considerable effort is underway to uncover the mechanisms that mediate adaptability. One well established mechanism for activity-dependent regulation of neurogenesis is the translation of network activity to newborn neurons using the neurotransmitter GABA (Ben-Ari et al., 2007; Ge et al., 2007a). Although GABA is the principal inhibitory neurotransmitter for mature neurons, GABA acts as a trophic factor for immature neurons and progenitor cells via Cl^−^-mediated depolarization. Here we review the dynamic role of GABA across GC development, highlighting the interneuron circuits that potentially serve distinct trophic and inhibitory functions. Although the focus is GABA_A_ receptor (GABAR)-mediated signaling, it is also relevant to note that dentate interneurons express a variety of other signaling molecules that influence adult neurogenesis that have been reviewed elsewhere (Masiulis et al., 2011).

## Diversity in GABA_A_ receptor-mediated signaling

There is substantial potential for diversity and specialization in signaling mediated by GABARs. Sources of diversity include the large number of distinct interneuron subtypes with differing contributions to network activity, flexibility in the polarity of GABAR- mediated postsynaptic potentials achieved via regulation of intracellular Cl^−^ concentration, the multiple modes of transmission mediated by tonic and phasic activation of GABARs, and numerous combinations of receptor subunits that can comprise receptors with differing GABA affinities and activation/inactivation kinetics. How newly generated GCs take advantage of the diversity in GABA signaling mechanisms at different developmental stages is just beginning to be understood. First we briefly describe these fundamental mechanisms.

### Diversity of GABAergic interneurons

GABAergic interneurons in the hippocampus comprise a heterogeneous population that can be classified by a variety of morphological, neurochemical, and physiological criteria (Maccaferri and Lacaille, 2003). An important criterion is based on the idea that interneuron functions are dictated by the specificity of their postsynaptic target-domain (Freund and Buzsaki, 1996). For example, axo–axonic and basket cells form synapses exclusively on the axon initial segments and proximal somatodendritic regions of principal cells, respectively, whereas numerous other interneuron subtypes target dendritic regions. It is well established that inhibition mediated by dendritic vs. perisomatic projecting interneurons differentially contribute to neural integration (Miles et al., 1996). Distinctive domain innervation allows single principal cells to take advantage of the overall diversity and specialization of interneuron functions by performing a variety of different computational tasks simultaneously in a spatially segregated manner (Klausberger and Somogyi, 2008). In the context of adult neurogenesis, the variety of interneuron subtypes raises questions about how these precise patterns of innervation are achieved, how the sequence of innervation contributes to the activity-dependent maturation of newborn cells, and how the innervation pattern contributes to the switch in the role of GABA signaling from an early trophic factor into complex regulator of neural timing and synchronization. In the simplest case, the laminar organization of interneuron axonal arborization is expected to determine the temporal sequence of synaptogenesis by interneuron subtypes, as dentate GCs born in the subgranular zone encounter increasing varieties of interneurons as newly formed neurites expand throughout the layers of the DG (Figure 1). Elucidating the precise sequence of GC innervation by interneuron subtypes will provide insight into how specific interneuron circuits subserve distinct functions during the course of GC maturation.

### The polarity of GABA_A_ receptor mediated responses

GABA released from interneurons acts at postsynaptic ionotropic GABARs that are permeable to Cl^−^, allowing shifts intracellular [Cl^−^] to alter the polarity of the postsynaptic response. Neuroprogenitors and immature neurons express high levels of the sodium-potassium-chloride exchanger NKCC1 that maintains high intracellular [Cl^−^] using sodium and potassium gradients. High intracellular [Cl^−^] results in GABAR-mediated depolarization since the Cl^−^ reversal potential E_[Cl^−^]_ is typically significantly more positive than the resting membrane potential (Ben-Ari, 2007). As maturation proceeds, up regulation of the potassium chloride coupled co-transporter KCC2 reduces intracellular [Cl^−^] to low mature levels, typically switching GABA responses from depolarizing to hyperpolarizing (Rivera et al., 1999). Sequential expression of NKCC1 and KCC2 is likewise thought to be responsible for the developmental shift in E_[Cl^−^]_ from approximately −40 mV in newborn GCs to more negative than −65 mV in mature GCs (Overstreet-Wadiche et al., 2005; Ge et al., 2006; Chiang et al., 2012; Sauer et al., 2012). The shift in the polarity of GABAergic responses in adult generated neurons occurs before the 4th week of maturation (Ge et al., 2006), and presumably is involved in the transition away from trophic functions that primarily rely on depolarization-mediated Ca^2+^ influx (Tozuka et al., 2005; Overstreet-Wadiche et al., 2006). However, the very negative resting membrane potential of GCs (below −75 mV) means that GABAR-mediated responses continue to depolarize even mature GCs (Chiang et al., 2012; Sauer et al., 2012). Depolarizing GABAR responses are typically inhibitory since the E_[Cl^−^]_ is hyperpolarized from the threshold for action potential generation and GABAR-mediated conductances shunt excitatory signals (Staley and Mody, 1992; Smith and Jahr, 2002), although modeling predicts that depolarizing GABAR responses in mature GCs could have excitatory actions depending on the exact timing and location of GABAergic postsynaptic currents (GPSCs) (Chiang et al., 2012).

### Multiple modes of GABAergic transmission

Whether depolarizing or hyperpolarizing, GABAR-mediated signaling occurs in two modes termed phasic and tonic (Farrant and Nusser, 2005). Phasic signaling refers to conventional synaptic transmission, in which GABA is released from presynaptic vesicles and activates postsynaptic GABARs on a rapid timescale. Tonic signaling refers to the activation of GABARs by ambient levels of GABA in the extracellular space. The different concentration profiles of GABA underlying synaptic and tonic signaling have significant consequences for GABAR function. The high (>1 mM) and brief (<1 ms) concentration profile in the synaptic cleft triggers rapid activation and deactivation of synaptic receptors (Mozrzymas, 2004), whereas persistent low concentrations of GABA favor receptor desensitization over activation (Overstreet et al., 2000). Despite accumulation of receptors in desensitized states, a large population of receptors exposed to low ambient [GABA] will equilibrate between desensitized, open and unbound states to generate steady-state conductances that can be large enough to affect excitability of dentate GCs (Overstreet and Westbrook, 2001; Nusser and Mody, 2002).

The source of GABA mediating phasic signaling is action potential-driven or spontaneous fusion of synaptic vesicles from presynaptic terminals, whereas the source of GABA mediating tonic signaling is less clear. Unlike extracellular levels of glutamate that are maintained in the low nM range by the 3:1 Na^+^: glutamate stoichiometry of glutamate transporters (Herman and Jahr, 2007; Tzingounis and Wadiche, 2007), the 2:1 Na^+^: GABA stoichiometry of GABA transporters predicts that a higher extracellular level of GABA (hundreds of nM) could persist in the absence of other sources of GABA release (Richerson and Wu, 2003; Farrant and Nusser, 2005). Ongoing synaptic release of GABA also contributes to the ambient concentration, potentially allowing regional and temporal regulation of extracellular tonic signaling to reflect ongoing network activity (Farrant and Nusser, 2005).

Synaptic vesicles containing GABA are released at morphologically identifiable presynaptic terminals with GABA acting at GABARs clustered at postsynaptic sites across the synaptic cleft. Thus phasic signaling is precisely localized to synapses although in some cases GABA diffusion outside the synapse can activate nearby extrasynaptic (perisynaptic) receptors (Kullmann, 2000). In contrast, it is not known whether there is regional specificity in the distribution of tonic GABAR currents across subcellular locations, although there is general agreement that tonic GABAR currents are primarily generated by extrasynaptic GABARs (see below).

Interestingly, a 3rd form of synaptic signaling that is intermediate between phasic and tonic signaling is used by specific subtypes of GABAergic interneurons (Szabadics et al., 2007). This form of transmission is mediated by the Ivy/Neurogliaform family of interneurons (Ivy/NG cells), and has recently been established to mediate slow inhibitory postsynaptic currents (IPSCs) sometimes termed GABA_Aslow_ (Capogna and Pearce, 2011). In contrast to the large and fast GABA transients produced at typical synapses made by perisomatic-projecting interneurons such as basket cells, GABA released from Ivy/NG cells presynaptic terminals generates a prolonged GABA transient with a low peak concentration that results in IPSCs with slow kinetics (Karayannis et al., 2010). The unusual GABA concentration transient could result from GABA released into the extracellular space from densely spaced neurogliaform presynaptic terminals, in a form of volume transmission (Olah et al., 2009; Capogna and Pearce, 2011).

### Heterogeneity of GABA_A_ receptor subunits

GABARs are heteropentameric channels typically composed of 2α and 2β subunits and either a γ or a δ subunit. Tonic and synaptic GABA signaling are associated with various GABAR subunits that have different biophysical properties and subcellular localizations [reviewed in Farrant and Nusser (2005)]. In dentate GCs, tonic signaling is mediated by extra- or peri-synaptically located α4, α5, and δ subunit containing GABARs (Stell et al., 2003; Chandra et al., 2006; Duveau et al., 2011). Conversely, α1, α2, and γ2 receptors are often clustered at synapses (Nusser et al., 1995; Sun et al., 2004) but consistent with the idea that subunits are not completely segregated these receptors are also found extrasynaptically. γ2 subunits are required for initial synaptic clustering and maintenance of GABARs within the synapse, and predominately found at synaptic sites (Essrich et al., 1998; Schweizer et al., 2003). Compensation and injury alters the expression levels and localization of subunits, suggesting flexibility in localization and function. For example, in epilepsy models there is altered expression and localization of δ and α2 subunits in dentate GCs that impair both tonic and phasic inhibition (Peng et al., 2002; Zhang et al., 2007; Rajasekaran et al., 2010). Furthermore, the subunit organizations of synaptic and non-synaptic GABAR clusters are dynamically regulated across development (Hutcheon et al., 2000, 2004).

## Trophic role of GABA in early development of dentate granule cells

The trophic function of GABA in neuronal development has been studied extensively in the neonatal brain. Pioneering work in the developing cortex showed that GABARs located on proliferating neuroprogenitor cells provide a depolarizing signal that increases intracellular calcium [(Ca^2+^)_i_] through the action of voltage-gated calcium channels (VGCCs), resulting in a reduction in DNA synthesis (LoTurco et al., 1995). Subsequent studies have established a common theme involving Ca^2+^ influx provided by GABAR-depolarization as a key component for many aspects of neuronal development, including proliferation, differentiation, and morphological maturation. The large literature establishing the role of GABA in neurodevelopment in both the developing and adult brain has been reviewed elsewhere (Ben-Ari et al., 2007; Ge et al., 2007a; Sernagor et al., 2010). Here we focus on reviewing recent studies delineating the contribution of specific GABARs and circuits to the development of adult generated dentate GCs.

Similar to principal cells in many developing brain regions, innervation by local GABAergic interneurons is established prior to glutamatergic synaptogenesis (Ambrogini et al., 2004; Esposito et al., 2005; Ge et al., 2006; Overstreet-Wadiche and Westbrook, 2006; Piatti et al., 2006), and the sequence of GABAergic innervation of newly generated cells follows the sequence established in the developing dentate where GPSCs with slow rise and decay kinetics are present prior to GPSCs with fast kinetics (Hollrigel and Soltesz, 1997; Liu et al., 1998; Esposito et al., 2005; Overstreet-Wadiche et al., 2005). Analogous to the synaptically “silent” immature neurons identified in the neonatal brain (Owens et al., 1999; Tyzio et al., 1999), neural stem cells and progenitors in the adult dentate also have tonic GABAR signaling prior to the formation of functional synapses.

### GABAR signaling in type I stem cell proliferation

**Figure 2:**
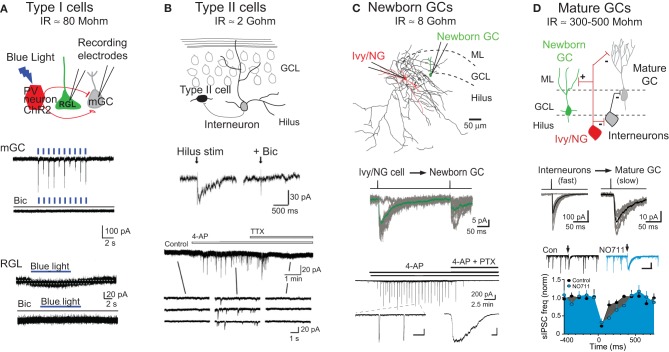
**Modes of GABAR signaling during GC development. (A)** Tonic signaling to Type I radial glial-like stem cells: Top, schematic depiction of whole cell patch clamp recording from mature granule cells (mGCs) or Type I GFP^+^ radial glial-like stem cells (RGLs) during photoactivation of PV^+^ interneurons expressing channelrhodopsin (ChR2). Middle, photoactivation of ChR2^+^PV^+^ interneurons (indicated by blue lines, 1 Hz) generates large synaptic currents in mature GCs that are blocked by the GABAR antagonist bicuculline (Bic). Bottom, photoactivation of ChR2^+^PV^+^ interneurons (blue bar, 8 Hz) generates small tonic currents in Type I RGLs that are blocked by bicuculline. Adapted from Song et al. (2012) with permission from Macmillan Publishers Ltd. Estimates for input resistance (IR) of Type I and Type II cells are from Fukuda et al. (2003). **(B)** Synaptic signaling to Type II neuroprogenitors: Top, schematic depiction of innervation of Type II cells by dentate interneurons. Middle, hilar stimulation evokes inward currents in Type II cells that are blocked by bicuculline. Bottom, the frequency of spontaneous activity in Type II cells is increased by bath application of 4-aminopyridine (4-AP; 100 μM), a potassium channel blocker that causes synchronous firing of dentate interneurons (Michelson and Wong, 1994; Markwardt et al., 2011). 4-AP-induced currents are blocked by tetrodotoxin (TTX). Insets show currents on an expanded time scale. Adapted from Tozuka et al. (2005) with permission from Elsevier. **(C)** Synaptic signaling to newborn GCs by Ivy/NG interneurons: Top, reconstructed Ivy/NG interneuron (red cell body and dendrites, black axon) from a paired recording with a POMC-GFP^+^ newborn GC (green). ML, molecular layer. Middle, current injection into presynaptic Ivy/NG cells evokes slow GPSCs in newborn GCs. The averaged unitary IPSC (green) is overlaid on individual currents (gray). From Markwardt et al. (2011). Bottom, bath application of 4-AP (100 μM) induces synaptic currents in newborn GCs that are blocked by picrotoxin (PTX). Insets show currents on expanded time scales [scale bars are 200 pA and 10 s (left) or 500 ms (right)]. From Markwardt et al. (2009). The estimate for IR of newborn GCs is from Overstreet et al. (2004). **(D)** Heterogeneous synaptic signaling to mature GCs: Top, schematic depiction showing that mature GCs are innervated by a variety of interneuron subtypes including Ivy/NG cells and perisomatic projecting interneurons. Ivy/NG cells also provide slow GABAergic inhibition to perisomatic-projecting interneurons. Middle, current injection in individual interneurons evokes either fast (left) or slow (right) uIPSCs in mature GCs, reflecting the heterogeneity of innervation. Bottom, spontaneous GPSCs in mature GCs arise from perisomatic-projecting interneurons (Soltesz et al., 1995). The frequency of sGPSCs is reduced by stimulation of the ML (arrows) that evokes slow NO711-sensitive IPSCs in mature GCs (insets) and perisomatic projecting interneurons (not shown). Together these results support the circuit diagram shown above. Scale in inset, 300 pA, 500 ms. Adapted from Markwardt et al. (2011). The estimate of IR for mature GCs is from Schmidt-Hieber et al. (2004) and Overstreet et al. (2004).

Radial glial cells (Type I cells) that express nestin are the putative neural stem cells of the adult DG (Seri et al., 2001; Bonaguidi et al., 2012). Located in the subgranular zone (Figures 1 and 2), Type I cells express the glial marker GFAP and have astrocytic properties including low input resistance and a resting potential near the K^+^ equilibrium potential (Filippov et al., 2003; Fukuda et al., 2003). Two reports indicate that Type I cells have functional GABARs that respond to high concentrations of exogenously applied GABA (Wang et al., 2005; Song et al., 2012), although GABA-evoked currents were not detected in another study (Tozuka et al., 2005). Type I cells do not have GABA synaptic innervation since both electrical stimulation and light-driven activation of parvalbumin (PV)-expressing interneurons fail to evoke synaptic currents (Figure 2A; Song et al., 2012). However, Song et al. (2012) proposed that tonic currents mediated by γ_2_-GABARs promote quiescence since knockout of the γ_2_ GABAR enhanced proliferation of Type I cells. Repression of stem cell proliferation by tonic GABAR signaling is likewise supported by the increased proliferation of progenitors seen in germline α_4_ GABAR knockout mice that have impaired tonic GABAR signaling in adult-generated progeny (Duveau et al., 2011). Tonic GABAR signaling in mature GCs is typically mediated by α_4_β_2_δ GABARs (Farrant and Nusser, 2005), but δ-GABAR subunit knockout mice did not have any of the deficits seen in the α_4_ knockout (Duveau et al., 2011). Thus, GABARs involved in tonic signaling in progenitors may be different than those of mature GCs. Indeed, γ_2_-GABAR subunits are typically associated with phasic GABAergic signaling since the γ_2_ subunit is essential for clustering receptors at synaptic sites (Essrich et al., 1998). It will be important to establish the mechanism by which tonic GABAR signaling through γ_2_ or α_4_ subunits contributes to stem cell proliferation, since the characteristics of Ca^2+^ influx generated by tonic depolarization of low-input resistance Type I cells will likely be distinct from Ca^2+^ transients generated by phasic GABAR signaling to later stage progenitors and newborn GCs that have high input resistance.

Interestingly, PV^+^ interneurons have a preferential role in promoting tonic GABAR signaling to Type I neural stem cells. PV^+^ interneurons form perisomatic basket-like synapses with mature GCs and have several functional specializations for rapid and precise phasic transmission (Bartos et al., 2001; Bucurenciu et al., 2008; Hu et al., 2010). Using the selective expression of light-activated channelrhodopsin (ChR2) in specific subtypes of interneurons, Song et al. (2012) showed that repetitive activation of PV^+^ expressing interneurons, but not somatostatin- or vasoactive intestinal peptide (VIP)-expressing interneurons, increased tonic GABAR currents in Type I cells in acute slices (Figure 2A). Furthermore, ChR2 activation of PV^+^ interneurons over a period of days *in vivo* reduced proliferation of Type I cells, whereas inactivation of PV^+^ cells with Halorhodopsin increased proliferation. Together these results suggest that despite the specializations the promote fast phasic GABA release from PV^+^ interneurons to mature GCs, PV^+^ interneuron activity regulates Type I stem cell quiescence via tonic GABAR signaling. The involvement of PV^+^ interneurons in proliferation is also supported by enhanced proliferation but reduced differentiation of progenitor cells when the BDNF receptor TrkB is selectively deleted from PV^+^ interneurons (Waterhouse et al., 2012). Thus PV^+^ interneurons have diverse roles GC function including modulation of proliferation via tonic signaling as well as control of mature GCs excitability by phasic signaling.

### GABAR signaling in type II progenitor differentiation

Type II cells comprise a heterogeneous population of progenitors that arise from Type 1 neural stem cells with variable mitotic capabilities and marker expression (Encinas and Enikolopov, 2008; Bonaguidi et al., 2012). Notably, the immature neuronal marker doublecortin is expressed in the “oldest” Type II progenitors. No longer displaying astrocytic properties, Type II cells are smaller than Type I cells with only minor processes, and they have high input resistance and heterogeneous voltage gated currents (Wang et al., 2005). Most Type II cells express functional GABARs (Tozuka et al., 2005; Wang et al., 2005), but robust tonic GABAR currents were not detected in nestin-GFP expressing Type II cells (Tozuka et al., 2005). Tonic GABAR currents are prominent, however, in adult generated cells at 3 days following retroviral infection (Ge et al., 2006), a time point when Type II progenitors are expected to comprise a large percentage of retroviral-labeled cells. Although tonic and phasic GABAergic signaling in distinct subtypes of Type II cells is not yet precisely defined, it is clear that during this period synaptic input are first detected in response to focal stimulation of the molecular layer and hilus (Figure 2B; Tozuka et al., 2005; Wang et al., 2005).

GABAergic signaling to Type II cells caused depolarization that increased [Ca^2+^]_i_ via voltage gated calcium channels (Tozuka et al., 2005). Treating cultured slices with GABA caused proliferating cells to differentiate, measured as in increase in *neuroD*, a gene required for granule cell differentiation. These results suggests that GABA-mediated depolarization drives Type II cells to leave the cell cycle and differentiate via the down regulation of anti-neuronal genes and up regulation of *neuroD*, establishing one mechanism underlying the so-called “excitation-neurogenesis” coupling previously identified by NMDAR activation of progenitors in culture (Deisseroth et al., 2004; Deisseroth and Malenka, 2005). GABAR mediated depolarization can also promote CREB signaling needed for cell survival. A large proportion of Type II progenitors undergo apoptosis, followed by a smaller proportion of cells undergoing apoptosis over the next 2–3 weeks after the transition to post mitotic newborn neuron (Sierra et al., 2010). Jagasia et al. (2009) demonstrated that cell autonomous loss of GABA depolarization leads to cell death of 1–2 week-old cells, an effect that is rescued by CREB signaling. Since selectively blocking tonic signaling in developing GCs by deletion of the α 4 GABAR subunit did not alter newborn neuron survival (Duveau et al., 2011), phasic rather than tonic GABAR signaling may be involved in promoting survival during the differentiation of Type II progenitors into newborn post mitotic GCs (see below).

The trophic role of GABA in GC maturation was elegantly established by knocking down NKCC1 expression in proliferating progenitors in order to block the depolarizing action of GABA throughout GC maturation (Ge et al., 2006). Two-week old newborn GCs that lack NKCC1 had shorter and less complex dendritic trees, and a delay in the development of glutamatergic synaptic inputs compared to 2-week-old control GCs. Although the mode of GABAR signaling and the developmental stage that were involved in these maturational delays were not specifically identified, subsequent work showed that deletion of α_4_-GABARs selectively blocks tonic signaling and impairs dendritic development in 2-week-old newborn GCs (Duveau et al., 2011). Thus early tonic GABAR signaling plays a role in dictating the rate of morphological maturation. On the other hand, deletion of the α_2_-GABAR subunit that is typically found in synapses disrupted dendritic structure in 1-month-old cells (Duveau et al., 2011). Together these results suggest that tonic and phasic signals have distinct and stage-specific trophic functions in Type II progenitors and newly post mitotic GCs.

### GABAR signaling to newly post mitotic GCs

Although little is known about the source and modes of GABAergic signaling to Type II progenitors, GABAergic signaling to newly post mitotic GCs has been extensively studied. Esposito et al. (2005) demonstrated that retroviral labeled newborn GCs between 1–3 weeks post-infection had evoked GPSCs with slow rise and decay kinetics similar to slow dendritic IPSCs observed in mature GCs and neurons in other brain regions. Evoked GPSCs with fast kinetics were only detected in retroviral labeled GCs that were ~4 week-old, suggesting that synapses mediating slow GPSCs develop prior to GABAergic synapses that mediate fast GPSCs generated at perisomatic locations. A GABAR antagonist applied to the dendritic region blocked slow evoked currents, supporting the dendritic origin for the earliest inputs (Esposito et al., 2005). Similarly, newborn GCs identified in proopiomelanocortin-GFP (POMC-GFP) reporter mice also received exclusively slow spontaneous and evoked GPSCs (Overstreet-Wadiche et al., 2005, 2006). These results suggest that early developing slow GABAR-mediated synaptic signaling could promote depolarization and Ca^2+^ influx needed for trophic functions, whereas later developing perisomatic synapses that mediate fast inhibitory currents may not be required to control neural output until after the development of excitatory glutamatergic synapses.

The mechanisms responsible for the early slow GABAR signaling in newborn GCs was studied in POMC-GFP reporter mice that allow identification of post mitotic newborn GCs with relatively uniform intrinsic excitable properties and morphology (Overstreet et al., 2004). This developmental stage is typically achieved at 10–12 days post-mitosis, when slow GABAR signaling is depolarizing and AMPA receptor-mediated transmission is not yet established (Overstreet-Wadiche et al., 2005, 2006). Markwardt et al. (2009) conducted a series of experiments to determine if slow synaptic currents resulted from non-specific spillover from nearby perisomatic terminals on mature GCs or dedicated inputs to newborn cells. GPSCs at perisomatic synapses have fast rise and decay times due to fast release mechanisms and the close proximity of postsynaptic receptors to the brief and high concentration of GABA within the cleft (Bartos and Elgueta, 2012), and are minimally affected by blockade of GABA transporters (Overstreet and Westbrook, 2003). However, the GABA transport antagonist NO711 robustly increased the amplitude, rise and decay times of GPSCs in newborn GCs, suggesting that the receptors underlying GPSCs were located far from the site of GABA release (Markwardt et al., 2009). Furthermore, impeding diffusion in the extracellular space enhanced GPSCs in newborn GCs but not fast GPSCs in mature GCs, suggesting that receptors on newborn GCs are far from saturation. Directly comparing the sensitivity of GPSCs to the low affinity antagonist TPMPA (Jones et al., 2001) confirmed that a lower concentration of GABA generated GPSCs in newborn GCs. These results are consistent with the possibility that slow GPSCs in newborn GCs result from spillover from perisomatic synapses on mature GCs, yet subsequent results ruled out this idea. First, a manipulation expected to reduce spillover transmission by reducing the density of active release sites did not preferentially decrease GPSCs in newborn GCs, and second, GPSCs in newborn GCs were enhanced by blockade of presynaptic GABA_*B*_ receptors whereas GPSCs in mature GCs were not, suggesting that the source of slow GPSCs in newborn GCs are unlikely the perisomatic terminals on mature GCs (Markwardt et al., 2009).

An alternative possibility to reconcile the results for and against spillover signaling was that slow GPSCs in newborn GC are generated by specific interneuron subtypes that mediate GABA_Aslow_ in the cortex, since GABA_Aslow_ also has characteristics of spillover even when it is generated by activation of a single interneuron (Szabadics et al., 2007). In support of this possibility, Markwardt et al. (2009) found coincident spontaneous slow GPSCs in simultaneous recordings from mature and newborn GCs, suggesting the existence of an interneuron subtype that mediates slow GPSCs in both newborn and mature GCs. This possibility was confirmed by the subsequent demonstration that single interneurons of the Ivy/NG family generate slow GPSCs in mature GCs (Armstrong et al., 2011) and newborn GCs (Figure 2C; Markwardt et al., 2011). Although almost all POMC-GFP labeled newborn GCs have slow evoked GPSCs, Markwardt et al. (2011) found that the probability of achieving a paired recording between a presynaptic interneuron and a newborn GC was very low compared to finding interneurons connected to mature GCs, likely reflecting the sparse innervation of newborn GCs. Furthermore, mature GCs received synapses from a wide variety of interneuron subtypes, whereas most (75%) of the presynaptic interneurons to newborn GCs had characteristics of Ivy/NG cells (the remaining presynaptic interneurons were not identifiable). Interestingly, bath application of the K^+^ channel blocker 4-AP generated low-frequency rhythmic firing in Ivy/NG interneurons resulting in phasic and tonic GABAR currents in newborn GCs (Figure 2C) that was correlated with inhibition in other interneuron subtypes (Markwardt et al., 2011). Further recordings from interneurons and mature GCs suggested that slow GABAR inhibition of other interneurons reduces spontaneous IPSCs in mature GCs (Figure 2D), demonstrating potential interactions between interneuron subtypes that mediate fast and slow inhibition. This idea is consistent with prior studies of GABA_Aslow_ in the CA1 (Capogna and Pearce, 2011), as well as the connectivity of Ivy/NG interneurons in the dentate molecular layer (Armstrong et al., 2011). A greater understanding of the interactions between specific interneuron subtypes will provide important insight into the complex roles of GABAergic circuits in controlling neurogenesis.

As mentioned above, synaptic GABAR signaling to newly post mitotic GCs could have a role in survival, since blockade of GABA depolarization at or prior to this stage reduces survival dependent on CREB activation (Jagasia et al., 2009), and there is an increase in GABA synaptic activity in newborn GCs associated with manipulations that enhance survival (Ambrogini et al., 2010; Chancey and Overstreet-Wadiche, unpublished). Recent results from our lab also suggest a novel role for slow GABAR signaling in providing the depolarization necessary for the activity-dependent incorporation of AMPA-type glutamate receptors at the first silent NMDAR-only synapses on newborn GCs (Chancey et al., 2012). These findings may provide experimental evidence supporting the longstanding idea that synaptic GABA depolarization allows synapse unsilencing at the first NMDAR-only glutamatergic synapses on developing neurons (Ben-Ari et al., 1997) and establish one specific mechanism whereby GABAR-mediated depolarization contributes to the functional integration of adult generated GCs (Ge et al., 2006).

## Inhibitory circuits control granule cell output

Together the studies described above demonstrate that GABA-mediated depolarization has trophic functions for all stages of granule cell development, from proliferation to synaptic integration. Early GABAergic signaling is associated with relatively slow and/or persistent activation of GABARs via ambient GABA in the extracellular space and a spillover-like mode of signaling from interneurons of the Ivy/NG family. Future work may determine how these forms of signaling are particularly suited to specific trophic functions. However, upon the development of glutamatergic synapses (represented by the transition from newborn GC to immature GC in Figure 1), inhibitory functions of GABA are likely required to control the output of immature GCs that retain high intrinsic excitability (Schmidt-Hieber et al., 2004; Esposito et al., 2005). Although many questions remain unanswered about the mechanisms and timing of the switch in trophic GABAergic signaling to the conventional inhibitory function, key events are the hyperpolarizing shift in the E_[Cl^−^]_ and the development of perisomatic synapses that mediate fast IPSCs. These two changes occur between 2–4 weeks post-mitosis, overlapping with the period when cortical afferents are establishing functional glutamatergic synapses (Esposito et al., 2005; Ge et al., 2006; Mongiat et al., 2009). Despite the significant differences between the intrinsic properties and synaptic connectivity of developing and mature GCs, both neonatal and postnatal derived GCs appear to ultimately converge into a functionally homogeneous population once full maturation is complete (Laplagne et al., 2006, 2007).

### Reduced inhibition of immature GCs promotes synaptic activation

Mounting evidence suggests that the physiological significance of adult neurogenesis derives from the distinctive, yet transient, properties of immature GCs that could endow them with a unique function in network activity. This idea has lead to intense interest in delineating the properties of immature GCs that are different from mature GCs, with an emphasis on intrinsic excitability and synaptic plasticity. The high input resistance of immature GCs results in large voltage responses produced from small membrane conductances, allowing action potential threshold to be achieved in response to small excitatory inputs (Schmidt-Hieber et al., 2004; Esposito et al., 2005; Mongiat et al., 2009). Immature GCs also display increased propensity for glutamatergic synaptic plasticity compared to mature GCs (Wang et al., 2000; Schmidt-Hieber et al., 2004; Ge et al., 2007b; Li et al., 2012). Since only a small fraction of dentate GCs fire action potentials in response to sensory stimulation in behaving rodents, the high excitability and plasticity of immature GCs could result in the preferential activation of immature GCs (Kee et al., 2007; Snyder et al., 2009; Alme et al., 2010), thereby allowing even a small population of adult generated GCs to contribute to network activity.

Recent findings directly demonstrate that immature GCs are more likely to fire action potentials in response to synaptic stimulation than mature GCs (Marin-Burgin et al., 2012). These authors used Ca^2+^ imaging and single cell recordings to show that stimulation of the perforant path triggers spiking in a greater fraction of immature GCs than mature GCs, and that immature GCs require a lower input strength (i.e., number of activated inputs) to elicit spiking. Enhanced activation of immature (4-week-old) GCs resulted from reduced inhibition relative to excitation, evidenced by the strong effect of GABAR blockade on activation of mature GCs but not immature GCs. The E_[Cl^−^]_ of immature GCs was nearly as hyperpolarized as E_[Cl^−^]_ of mature GCs, indicating the polarity of GABAR responses was not responsible for reduced inhibition, and the peak ratio of excitation to inhibition was also similar. However, the rise time of perisomatic-evoked IPSCs in immature GCs was slower than the rise time of perisomatic IPSCs in mature GCs, resulting in a higher ratio of excitation to inhibition at the time of spike initiation (Marin-Burgin et al., 2012). These results demonstrate that the slow kinetics of IPSCs contributes to greater synaptic activation of immature GCs. Whether slow kinetics resulted from differences in relative innervation by perisomatic projecting PV^+^ and CCK^+^ basket cells that differ in their speed of signaling (Bartos and Elgueta, 2012) or developmental differences in postsynaptic GABAR subunits (Hollrigel and Soltesz, 1997) will be interesting to test. Notably, these results reveal that the precise properties of interneuron signaling to immature GCs have important consequences for determining their role in network activity.

### Powerful inhibitory circuits maintain sparse activation of mature GCs

The important role of GABAR signaling across all stages of GCs development is perhaps not surprising, given the well-established role of synaptic inhibition in the “gating function” of the DG. Historically the dentate has been viewed as a filter or gate that restricts the flow of neural activity through the hippocampus, particularly in the context of pathologies that promote epileptogenesis (Dudek and Sutula, 2007). The DG has also received considerable attention for its physiological function in the computational task of pattern separation, or the ability to differentiate similar but different patterns of spatial or sensory inputs (Stella et al., unpublished; Vivar et al., unpublished). Both dentate gating and pattern separation require sparse activation of the numerous GCs and accordingly, physiological stimuli in behaving rodents activates only small subsets of dentate GCs (Jung and McNaughton, 1993; Chawla et al., 2005; Alme et al., 2010; Neunuebel and Knierim, 2012). Sparse activation is also apparent *in vitro* by minimal activation of GCs in response to afferent input from the perforant path; however widespread activation results even with incomplete blockade of GABARs (Coulter and Carlson, 2007). The central role of inhibitory circuits in the formation of granule cell place fields has been modeled as a competitive network phenomenon that engages strong feedback inhibition to silence the majority of GCs (de Almeida et al., 2009; Renno-Costa et al., 2010), consistent with strong activity-induced inhibition identified *in vivo* (Sloviter, 1991). *In vitro* work also demonstrates that perforant path stimulation preferentially recruits fast spiking interneurons that control the output of GCs in a frequency-dependent manner (Ewell and Jones, 2010). Numerous types of interneurons innervate mature GCs, including those defined by the location of their somato-dendritic and axonal domains such as molecular layer perforant path-associated (MOPP) cells and hilar commissural-association (HICAP) cells [reviewed by Houser (2007)], as well as interneurons defined by their physiological properties and neurochemical content such as PV^+^ and CCK^+^ basket cells (Hefft and Jonas, 2005), somatostatin-expressing cells (Zhang et al., 2009) and nitric-oxide expressing neurogliaform cells (Armstrong et al., 2011). It would be interesting to determine how specific interneuron subtypes contribute to sparse activation of GCs and behavioral performance of tasks that require information processing by the DG.

## Conclusions

GABA-mediated signaling is involved in all stages of dentate GC maturation and function. Early in GC development, slow forms of GABA-mediated depolarization provide trophic signals necessary for regulating proliferation, differentiation, survival, and synaptic integration. Then during a transient time period between GABAergic excitation and the full development of fast synaptic inhibition, reduced inhibition contributes to the high responsiveness of young GCs to cortical inputs. Once GCs become fully mature, fast and powerful synaptic inhibition shapes GC output, allowing for the sparse population coding necessary for DG functions including pattern separation. Specific subtypes of interneurons, modes of GABAR activation, and GABAR subunits serve distinct roles in each stage of GC development, illustrating the diversity of GABAR signaling mechanisms and also raising many additional unanswered questions. For example, it is not clear what controls the sequence and timing of innervation by all the various DG interneuron subtypes, nor the timing of the switch in GABA signaling from trophic depolarization to predominantly inhibition. There is also very little known about postsynaptic GABA_B_ receptor signaling, despite evidence that neurogenesis can be altered by blockade of GABA_B_ receptors (Felice et al., 2012). Immature GCs, however, do express functional presynaptic GABA_B_ receptors that modulate presynaptic excitability (Cabezas et al., 2012). Finally, the extent to which GABAergic mechanisms contribute to regulation of neurogenesis in specific pathological or physiological conditions is just beginning to be explored (Li et al., 2009; Sun et al., 2009; Song et al., 2012). Future studies will provide new insights into how the diversity of GABAR mediated signaling in the DG contributes to dynamic regulation of GC development and excitability.

### Conflict of interest statement

The authors declare that the research was conducted in the absence of any commercial or financial relationships that could be construed as a potential conflict of interest.
